# *Leishmania infantum* EndoG Is an Endo/Exo-Nuclease Essential for Parasite Survival

**DOI:** 10.1371/journal.pone.0089526

**Published:** 2014-02-26

**Authors:** Eva Rico, Cristina Oliva, Kilian Jesús Gutierrez, Juan Fernando Alzate, Carlos Mario Genes, David Moreno, Elena Casanova, Alba Gigante, María-Jesús Pérez-Pérez, María-José Camarasa, Joachim Clos, Federico Gago, Antonio Jiménez-Ruiz

**Affiliations:** 1 Departamento de Biología de Sistemas-Unidad Asociada al Consejo Superior de Investigaciones Científicas (CSIC), Universidad de Alcalá, Alcalá de Henares, Madrid, Spain; 2 Departamento de Microbiología y Parasitología, Facultad de Medicina, Universidad de Antioquia, Medellín, Colombia; 3 Instituto de Química Médica - Consejo Superior de Investigaciones Científicas (IQM-CSIC), Madrid, Spain; 4 Bernhard Nocht Institute for Tropical Medicine, Hamburg, Germany; 5 Departamento de Ciencias Biomédicas - Unidad Asociada al Consejo Superior de Investigaciones Científicas (CSIC), Alcalá de Henares, Madrid, Spain; Federal Institute for Vaccines and Biomedicines, Germany

## Abstract

EndoG, a member of the DNA/RNA non-specific ββα-metal family of nucleases, has been demonstrated to be present in many organisms, including Trypanosomatids. This nuclease participates in the apoptotic program in these parasites by migrating from the mitochondrion to the nucleus, where it takes part in the degradation of genomic DNA that characterizes this process. We now demonstrate that *Leishmania infantum* EndoG (LiEndoG) is an endo-exonuclease that has a preferential 5′ exonuclease activity on linear DNA. Regardless of its role during apoptotic cell death, this enzyme seems to be necessary during normal development of the parasites as indicated by the reduced growth rates observed in *LiEndoG* hemi-knockouts and their poor infectivity in differentiated THP-1 cells. The pro-life role of this protein is also corroborated by the higher survival rates of parasites that over-express this protein after treatment with the LiEndoG inhibitor **Lei49**. Taken together, our results demonstrate that this enzyme plays essential roles in both survival and death of *Leishmania* parasites.

## Introduction

Members of the Endonuclease G (EndoG) family have been found in all organisms whose genomes have been fully sequenced. One of the most important roles described for these enzymes is their participation in the apoptotic process whereby EndoGs translocate to the nucleus and contribute to the degradation of genomic DNA into oligonucleosomal fragments [1]. All of these proteins belong to the superfamily of non-specific ββα-metallonucleases [2] and their nuclease activity depends on the presence of divalent cations such as Mg^2+^, Mn^2+^ or Co^2+^ whereas it is inhibited by moderate concentrations of monovalent cations such as K^+^ or Na^+^ [2], [3], [4]. EndoGs are encoded by a nuclear gene and imported into the mitochondrion as a consequence of the presence of an N-terminal signal peptide [5]. Mitochondrial localization is essential because its ectopic expression in the cytosol induces cell death [2].

Despite the similarities among EndoG family members, their nuclease activities are not identical: yeast and *Neurospora* enzymes contain an intrinsic 5′ exonuclease activity that is absent in their mammalian counterparts [6]. In fact, mammalian mitochondria also contain EXOG, an endo-exonuclease that is considered to have originated from the duplication of an ancestral nuclease gene that generated the paralogous EndoG- and EXOG-protein subfamilies in higher eukaryotes. This way, the full endo/exonuclease activities found in mitochondria of lower eukaryotes were maintained [7].

Mammalian members of this family of nucleases attack the nucleotide sequence in duplex DNA in a highly nonrandom fashion and show a particularly strong, but not exclusive, preference for nicking at positions adjacent to guanines [8]. This striking preference to attack guanine tracts is the hallmark of the endonuclease activity of the mammalian members of this family and the reason why these enzymes were named Endonucleases G. In contrast, the yeast endo-exonuclease has no preference to nick specific sequences in double-stranded DNA (dsDNA) [6].

Apart from their involvement in the death process, a pro-life role for these nucleases has also been suggested in several organisms. Thus, reduction in the levels of the *C. elegans* EndoG ortholog cps-6 by RNA interference (RNAi) delays cellular growth during development of this worm [1]. Similarly, deletion of the yeast EndoG ortholog *Nuc1p* sensitized cells to cell death using standard culture conditions but, on the contrary, diminished apoptotic death when mitochondrial respiration was increased. These findings point to a dual (pro-life and pro-death) role [9].

In this work we show that *Leishmania infantum* EndoG (LiEndoG) not only displays endonuclease activity but, similarly to its counterparts from yeast and *Neurospora*, also functions as a 5′-exonuclease. In an attempt to evaluate its possible pro-life role, we used a gene replacement strategy to diminish endogenous LiEndoG levels. This decrease reduced the growth rate of the parasites and limited their capacity to infect and survive inside differentiated THP-1 cells. Moreover, results from assays on control and *LiEndoG*-transfected parasites using a recently described LiEndoG inhibitor support the vital function of this protein.

## Materials and Methods

### Cells and culture conditions

*L. infantum* promastigotes (M/CAN/ES/96/BCN150 MON-1), kindly provided by Dr. Alonso (CBMSO- Universidad Autónoma Madrid, Spain), were grown in RPMI-1640 medium (Gibco, Paisley, UK) supplemented with 10% heat-inactivated fetal calf serum (FCS), antibiotics, and 25 mM HEPES at 26°C. Parasite death was induced by addition of edelfosine (Calbiochem) or **Lei49** (2-[(S)-2-Amino-3-methylbutanoyl-oxy]ethyl [(2R,3S,5R)-5-(thymin-1-yl)-2-(trityloximethyl)tetrahydrofuran-3-yl] succinate) to the culture medium [10]. Growth curves were plotted as the product of cell concentration versus time. The graph shows the log_2_ values of the parasite concentration. Parasites were kept at the log phase by daily dilution to 1.1×10^6^–1.5×10^6^ parasites/mL and successive dilutions were considered to calculate the number of parasites.

### *S*equence cloning and construct design

DNA fragments containing the *LiEndoG* 5′UTR were obtained after amplification of genomic DNA with 5′GGGGAATTCTGAACATCACAGTGTGGAG3′ and 5′CTCATGGGTACCTGATGAATCGTTTCGCGCA3′ primers (underlined sequences highlight the EcoRI and the KpnI restriction sites respectively). DNA fragments containing the LiEndoG 3′UTR were obtained after amplification of genomic DNA with 5′GGGCTGCAGCTTGTCGCGGTTTTGG3′ and 5′GGGGGATCCTTGGGGTTGCCCTCGTTGC3′ primers (underlined sequences highlight the PstI and the BamHI restriction sites respectively). Gene replacement was achieved by transfection of the parasites with DNA constructions containing the *LiEndoG* 5′ UTR and 3′UTR ends (cloned using the EcoRI/KpnI and BamHI/PstI restriction sites, respectively) within the pUC19 vector containing either the Puromycin *N*-acetyl-transferase (PAC) (pUC19*5′UTRLiendoG:puro:3′UTRLiendoG*) or the neomycin resistance gene (*neo*) (pUC19*5′UTRLiendoG:neo:3′UTRLiendoG*). Both *pac* and *neo* genes had been previously inserted into the pUC19 vector using the KpnI and BamHI restriction sites.

### EndoG expression and purification

*E. coli* BL21(DE3) pLys bacterial cells were transformed with pRSET-*LiendoG*. Recombinant LiEndoG (rLiEndoG) expression was then induced during 30 minutes with 1 mM IPTG at 37°C. Because most of the expressed protein was found in the non-soluble fraction, *E. coli* cells were lysed in 6 M GuHCl and the cleared lysate was loaded onto a Ni-NTA resin under denaturing conditions according to the manufacturer's instructions (Qiagen). On-column refolding of the protein was achieved by progressive reduction of the urea concentration from 6 M to 1 M. The protein was used for monoclonal antibody production according to standard protocols [11].

### Nuclease activity assay

1 µg of plasmid DNA (pRSETa) was digested in a final volume of 20 µL with increasing amounts of rLiEndoG (20–150 ng) for 1 h at 37°C. Digested DNA samples were denatured using 0.1 volumes of NaOH 1 M, heated 2 minutes at 55°C, neutralized with 0.2 volumes of TrisHCl 1 M, pH 4.0, chilled in ice and finally diluted in Milli-Q water in order to avoid the effect of the salts on the migration of the samples during the subsequent electrophoresis. The DNA samples were finally analyzed on an agarose gel at 1.2% w/v, stained with ethidium bromide and visualized under UV light.

Nuclease activity was also tested against a 500-bp PCR product amplified with a forward primer fluorescently labeled at its 5′ end with 6-carboxyfluorescein (FAM) (Applied Biosystems). The PCR fragment with a fluorescent 5′ end was purified using a spin-column (Qiagen) in order to remove the primers used for the amplification. The PCR fragments were digested in a final volume of 20 µL with increasing amounts of rLiEndoG or DNase I (Boehringer Mannheim) at 37°C for 1 h. 5 µL of the digested samples were heat-denatured and diluted 1∶10 and analyzed by capillary electrophoresis using the 3130 Genetic Analyzer (Applied Biosystems). The results obtained were examined using the Peak Scanner v1.0 (Applied Biosystems) software. The remaining 15 µL from each digestion were analyzed by electrophoresis on an agarose gel at 1.2% w/v, stained with ethidium bromide and visualized under UV light.

### Promastigote transfection

The parasites were harvested in logarithmic growth phase and transfected by electroporation as previously described [12]. Stably transfected strains were selected in RPMI/20%FCS with 20 µg/mL of puromycin for hemi-KO parasites and with 20 µg/mL of puromycin and 30 µg/mL of G418 for double-KO parasites.

The pUC19*5′UTRLiendoG:puro:3′UTRLiendoG* construct was linearized using the restriction enzymes HindIII and EcoRI. The pUC19*5′UTRLiendoG:neo:3′UTRLiendoG* construct was linearized using the restriction enzyme EcoRI. The digestion products were gel purified (Illustra GFX gel purification kit, General Electric) prior to transfection. Individual clones were isolated in agar plates [13]. Cultured cells were diluted 1∶5 when the density exceeded 5×10^6^ cells/mL and cell densities were determined by triplicates using cell counting grids (Marienfeld, with a depth of 0.1 mm).

### Protein Extraction and Western Blotting

40×10^6^ parasites were lysed in 100 µL of Laemmli lysis buffer and boiled for 10 minutes. 20 µL of each sample were used for SDS-PAGE analysis in 10% acrylamide gels. For Western blot analysis, proteins were transferred from the gels to a PROTRAN nitrocellulose membrane (Whatman) in transfer buffer (25 mM Tris-HCl, 192 mM glycine, 20% methanol, 0.02%SDS, pH 8.3). The membranes were first incubated for 2 h in a blocking solution consisting of TBS-T buffer (150 mM NaCl, 10 mM Tris, 0,1% Tween, pH 8) supplemented with 5% of BSA (bovine serum albumin) (Sigma) and then with anti-LiEndoG monoclonal antibody for 16 hours at 4°C with shaking. An anti-mouse monoclonal antibody HRP-conjugated (Sigma) diluted 1∶5000 in the blocking solution was used as the secondary antibody.

Polyclonal anti-Hsp70 antibody diluted 1∶2000 was used as a loading control. In this case, a polyclonal anti-rabbit antibody conjugated with HRP (Santa Cruz) diluted 1∶5000 was used as the secondary antibody. Antibodies were recognized with ECL reagent (Thermo Scientific).

### THP-1 infections

THP-1 cells were seeded at 120.000 cells mL^−1^ in 24-multiwell plates (Nunc, Roskilde, Denmark) and differentiated to macrophages for 24 h in 1 mL RPMI-1640 medium containing 10 ng mL^−1^ phorbol 12-myristate 13-acetate (PMA, Sigma–Aldrich) at 37°C. Then the culture medium was removed and *L. infantum* promastigotes in a ratio 20∶1 to THP-1 cells were added in RPMI-1640 medium. After 24 hours of incubation all medium with non-infecting promastigotes was removed. Cells were washed three times with 1× phosphate-buffered saline (PBS) and detached with TrypLE Express (Invitrogen, Leiden, The Netherlands) according to the manufacturer's indications. Infection rates were measured by flow cytometry as the percentage of GFP positive cells. The number of amastigotes inside the cells was indirectly determined based on the intensity of the green fluorescence of the infected cells.

### Mitochondrial transmembrane potential determination

Logarithmically growing promastigotes (1×10^6^ cells) were incubated with Mitotracker Red CMXRos (Life Technologies) to a final concentration of 100 nM and maintained under normal growing conditions for 45 minutes. As a mitochondrial membrane depolarization control the protonophore carbonyl cyanide m-chloro phenyl hydrazone (CCCP) was used to a final concentration of 100 µM under the same conditions. Subsequently, samples were analyzed using a FC500 MPL flow cytometer (Beckman-Coulter).

### LiEndoG activity

The nuclease activity of LiEndoG in the presence of **Lei49** was monitored by measuring the increase in fluorescence derived from the digestion of a piece of dsDNA. 6-Carboxyfluorescein (FAM)-derived fluorescence is quenched by the proximity of tetramethylrhodamine (TAMRA) in the undigested probe constructed by hybridization of the oligonucleotides FAM-5′-CTG TCG CTA CCT GTG G-3′-TAMRA and FAM-5′- CCA CAG GTA GCG ACA G-3′-TAMRA. Digestion of any of the two oligonucleotides causes separation of the fluorophore from the quencher giving rise to emission of a fluorescent signal. 30 pmol of the double-stranded probe were digested with 2.5 ng mL^−1^ LiEndoG. Reactions were monitored in a Victor 1420 Multilabel Counter (Wallac) at an excitation and emission wavelengths of 492 and 517 nm, respectively.

### Cell viability assay

Drug treatment of promastigotes was performed during logarithmic growth phase at a concentration of 2×10^6^ parasites/mL at 26°C. The percentage of living cells was evaluated by flow cytometry by the propidium iodide (PI) exclusion method [14]. Briefly, treated parasites were stained for 10 min with 5 µg/mL PI. The number of PI-negative parasites was determined in a Beckman Coulter FC500 flow cytometer.

### Confocal microscopy

All experiments were done starting with 10×10^6^ logarithmically growing promastigotes and visualized under a LEICA TCS-SP5 confocal microscope. Mito-Tracker Red CMXRos (Life Technologies) was used for mitochondrial localization at a final concentration of 200 nM according to the manufacturer's instructions. Briefly, cell cultures were centrifuged to obtain a cell pellet and gently suspended in warm (26°C) staining solution containing 200 nM Mito-Tracker Red CMXRos. After 45 minutes at 26°C the cells were re-pelleted by centrifugation and suspended in fresh warm medium for 30 minutes. Finally, the cells were re-pelleted again and washed in PBS. Cells were then fixed in 2% paraformaldehyde in PBS for 10 minutes at room temperature and washed with PBS. 1.5×10^6^ cells were spread on poly-lysine pre-coated slides and permeabilized with 0.1% Triton X-100 (TX-100) in PBS solution for 10 minutes. Preparations were then incubated with: i) blocking solution (2% BSA, 0.1% TX-100 in PBS) for 60 minutes at room temperature, ii) primary monoclonal antibody anti-LiEndoG (diluted 1∶2 in blocking solution) for 16 h at 4°C, and iii) antibody Alexa Fluor 488 anti-mouse (diluted 1∶1000 in blocking solution) (Life Technologies). Each incubation was followed by 3 washes with 1×PBS.

Finally, the samples were covered with ProLong Gold Antifade Reagent with DAPI liquid mountant (Life Techologies) and visualized under the microscope.

### Statistics

All the analyses were carried out with a minimum of three independent experiments. The statistical significance of the differences between treatments was evaluated by a two-tailed unpaired t-test.

## Results

### The endonuclease activity of LiEndoG generates single-stranded cuts in the DNA double helix

**Figure 1:**
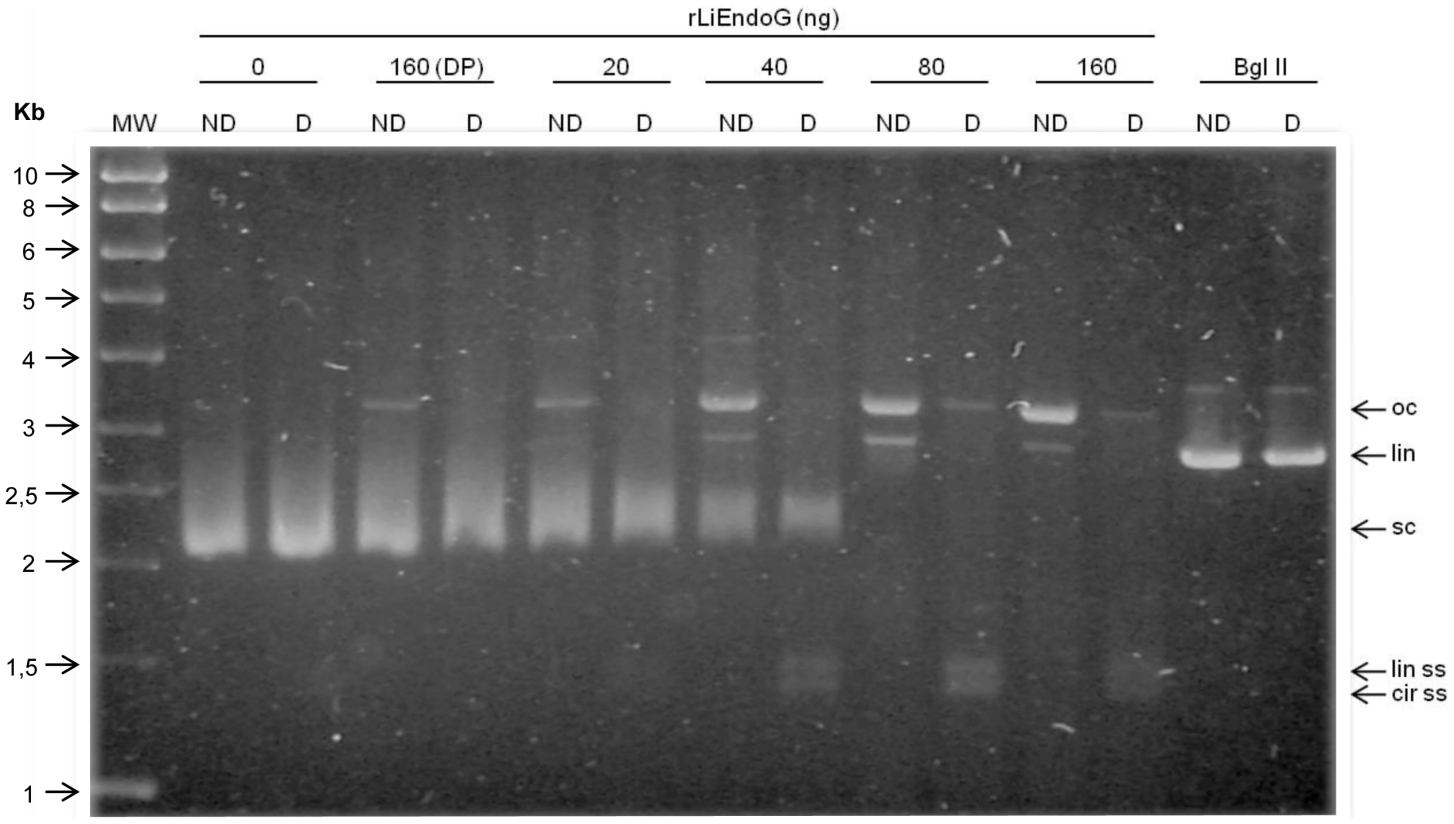
Analysis of rLiEndoG activity on double-stranded DNA. 1 µg of supercoiled plasmid DNA (sc) was digested with increasing amounts of rLiEndoG (0, 20, 40, 80 and 160 ng) and with 160 ng of denatured rLiEndoG protein (160DP) as a negative control. The restriction enzyme Bgl II (Bgl II) was used as a control to generate linear DNA (lin). Each sample of digested DNA was split into two aliquots and one of them was heat-denatured (D) while the other one remained non-denatured (ND). All samples were electrophoresed in a 1% agarose gel and visualized under UV light. The different forms of plasmid DNA are indicated: open circular (oc); linear double-stranded (lin); supercoiled (sc); linear single-stranded (lin ss); circular single-stranded (cir ss).

To determine whether, as described for other endonucleases [6], LiEndoG is able to produce nicks in dsDNA, we proceeded to digest supercoiled (sc) plasmid DNA with increasing amounts of rLiEndoG expressed in *E. coli*. Each digested sample was divided into two aliquots, and one of them was denatured to follow the migration of the distinct forms of released single-stranded DNA. The restriction enzyme Bgl II was used as a control for the simultaneous digestion of both strands to generate a linear (lin) DNA molecule (Figure 1; Bgl II). rLiEndoG is able to digest supercoiled closed circular DNA, thus demonstrating that this enzyme displays endonuclease activity. The enzyme primarily generates open circular (oc) intermediates while only a very faint band of linear (lin) DNA can be observed. Denaturation of the samples (D lanes) allowed the detection of the fully-sized linear and circular single-stranded (ss) DNA molecules that made up the open circular forms generated after digestion. Because ethidium bromide has much higher affinity for dsDNA, these ss molecules are weakly stained compared to the oc DNA. Accordingly, rLiEndoG displays endonucleolytic activity but digests only one of the two strands of the double helix in every digestion step. However, this enzymatic activity may render linear DNA as a minor digestion product when two independent cuts in the complementary strands are so close together that base pairing can no longer maintain the circular structure (Figure 1; lin). The progressive decrease in the total ethidium-derived fluorescence reveals a process of DNA degradation that can be explained by the release of small oligonucleotides from the double helix after accumulation of single strand breaks. Thus, the enzyme seems to cut stochastically any of the two strands, as has been described for *Drosophila melanogaster* EndoG [15].

### rLiEndoG shows exonuclease activity

**Figure 2:**
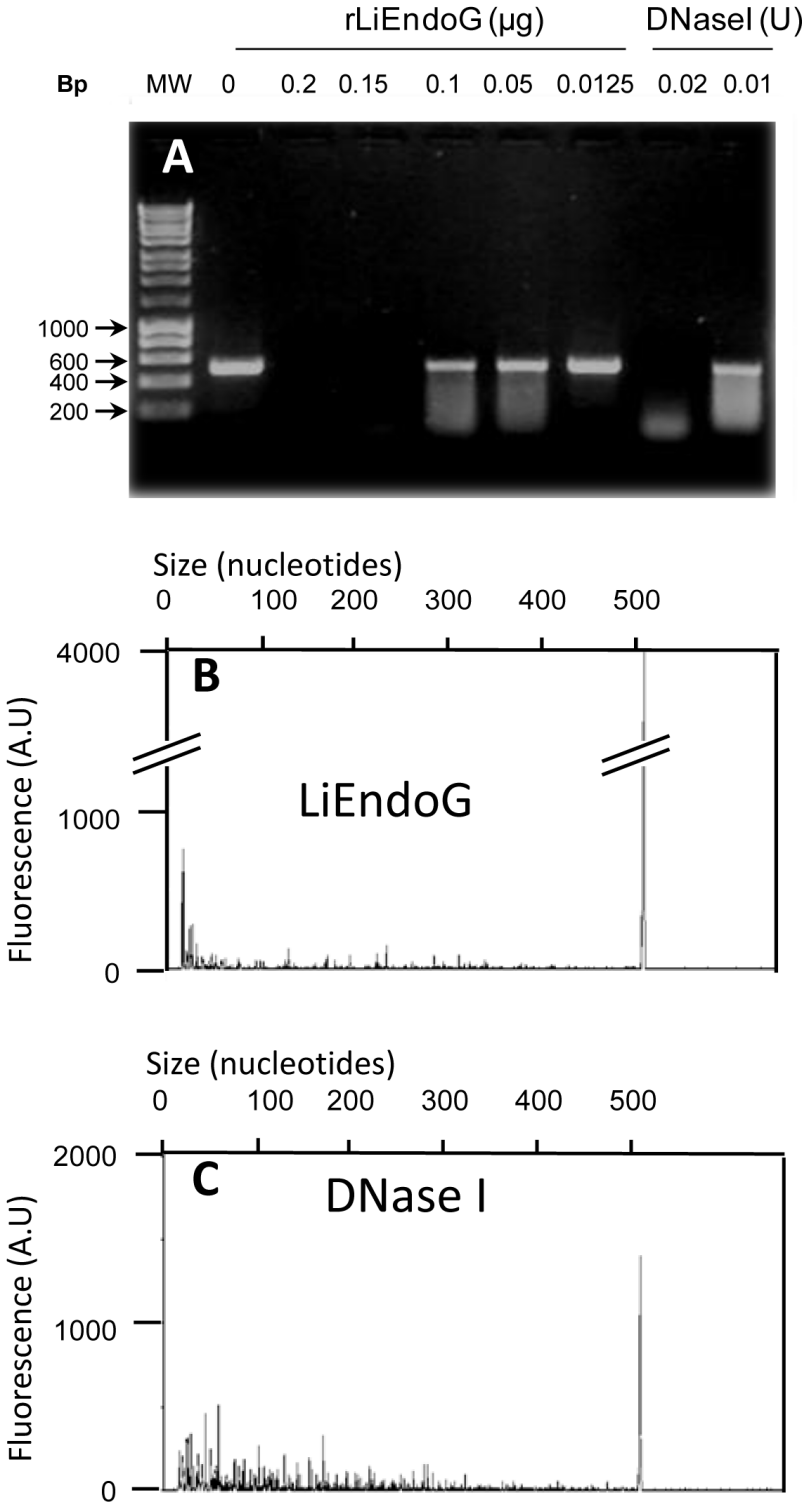
Exonuclease activity of rLiEndoG. 500′-labeled with FAM were digested with rLiEndoG or DNase I. The amount of enzyme used for digestion is indicated. Digestion products were processed by both agarose gel (1%) and capillary electrophoresis. **A**) Agarose gel of the DNA fragments generated after 1 h of digestion with rLiEndoG or DNase I. **B**) Capillary electrophoresis of the DNA probe digested for 1 h with 0.1 µg of rLiEndoG. **C**) Capillary electrophoresis results obtained for the DNA probe digested for 1 hour with 0.01 units of DNase I. Digested DNA was heat-denatured prior to capillary electrophoresis. Fluorescence intensities (arbitrary units) of the single-stranded DNA fragments generated after digestion and denaturation are shown on the y axis. Sizes of the ssDNA fragments (in nucleotides) are shown on the x axis. Fragment sizes were analyzed with the Peak Scanner (Applied Biosystems) software. Accurate sizes can only be predicted for fragments longer than 50 nucleotides.

While EndoGs from higher eukaryotes display only endonuclease activity [6], Nuc1p from *S. cerevisiae* behaves both as an endonuclease and as an exonuclease [16]. Once established that LiEndoG is an endonuclease, we addressed the possibility that it might also display exonuclease activity. To this end, genomic DNA was amplified by PCR using a FAM-labeled forward primer so as to generate a PCR product with a fluorescent 5′ end. The resulting PCR product was digested with increasing concentrations of rLiEndoG. DNase I was used as a control for endonuclease activity [17], [18]. The products resulting from the digestion were processed by both agarose gel electrophoresis (Figure 2A) and capillary electrophoresis (Figure 2B and 2C). Whereas gel electrophoresis allows the detection of all the dsDNA fragments generated after digestion, only ss FAM-labelled fragments (fragments containing the labelled 5′ end) are detected in capillary electrophoresis.

Digestion with rLiEndoG mostly generated fluorescent low-molecular-weight ss- DNA fragments, being the major peak the one closest to the y axis (Figure 2B). This accumulation of small DNA fragments is not caused by extensive digestion because the conditions of the reaction were adjusted to allow the detection of a major peak of undigested DNA (500 bases). By contrast, the peak pattern observed after digestion with DNase I is much more dispersed along the x axis and the smallest fragments are not the most abundant (Figure 2C). Taken these results together, the nicking activity of rLiEndoG appears to be preferentially directed against the 5′ end of DNA.

### LiEndoG has a pro-life role

**Figure 3:**
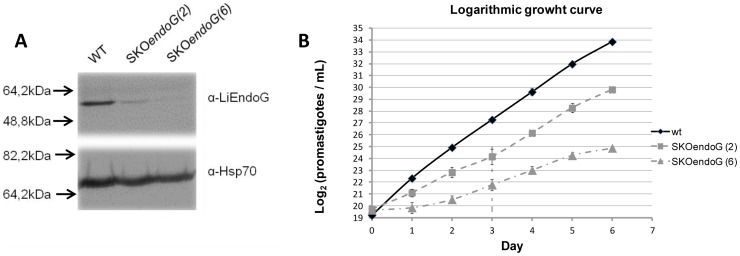
LiEndoG single allele replacement reduces L. infantum promastigotes growth rate. **A**) Immuno-blot using the monoclonal antibody α-rLiEndoG against cellular extracts from wild-type (WT) promastigotes and from two clones of *L. infantum* promastigotes with a single copy of the *LiEndoG* gene: SKO*endoG*(2) and SKO*endoG*(6). A Polyclonal α-Hsp70 (1∶5000) antibody was used as a loading control. **B**) Logarithmic growth curve for WT, SKO*endoG*(2) and SKO*endoG*(6) promastigotes. The curve is expressed as the log_2_ parasite number per mL. Error bars represent standard deviations; results are representative of three independent experiments.

EndoGs from other lower eukaryotes have been reported to play a dual role in both cell death and survival [9]. A gene replacement strategy was followed to determine whether LiEndoG is also involved in pro-life roles in *Leishmania*. All attempts to delete both *LiEndoG* alleles were unsuccessful but parasites remained viable after single allele replacement (*LiEndoG* ^−/+^). The results shown in Figure 3 indicate that LiEndoG expression is highly reduced in the *LiEndoG* ^−/+^ promastigotes (Figure 3A). Growth of these parasites was analyzed by counting the number of cells for 6 consecutive days. Parasites were kept in the logarithmic growth phase by daily dilutions (Figure 3B). Since the y axis represents the log_2_ of the number of parasites an increase of one unit denotes one round of division of the entire population. Results for two different randomly selected *LiEndoG* ^−/+^ clones (SKOendoG(2) and SKOendoG(6)) are shown. *LiEndoG^−/+^* promastigotes grow at a much lower rate than do their wild-type counterparts under optimal growth conditions. In fact, a difference of 9 log_2_ units between clone 6 and the wild-type parasites is apparent after 6 days of culture. Variations in the growth rates of clones 2 and 6 seem to correlate with differences observed in the residual levels of expression of LiEndoG. Thus, the disadvantage of the parasites harboring reduced levels of LiEndoG is consistent with a pro-life role for this protein.

**Figure 4:**
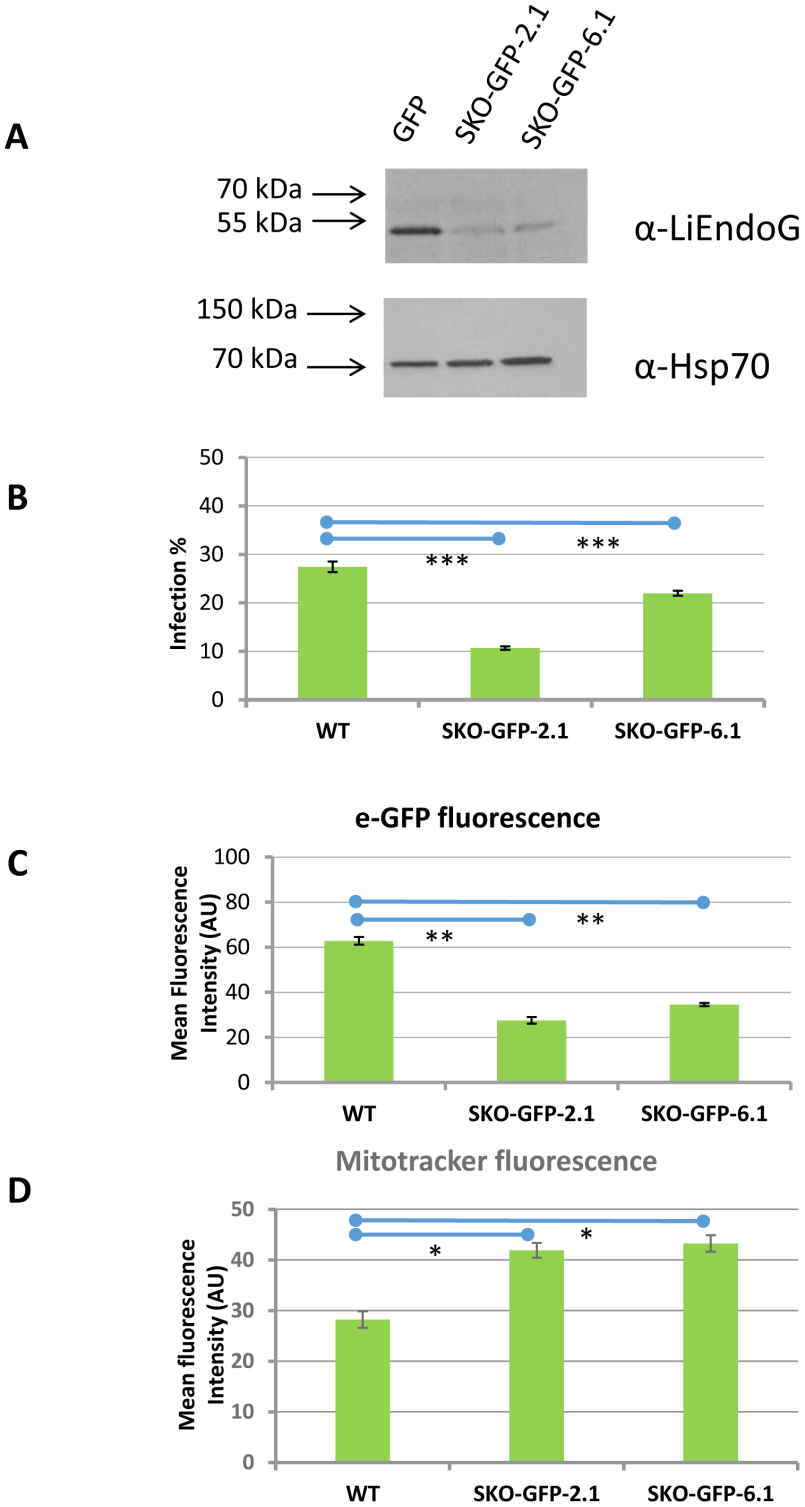
LiEndoG single allele replacement impairs macrophage infection, reduces intracellular survival and increases mitochondrial transmembrane potential. **A**) Immuno-blot using the monoclonal antibody α-rLiEndoG against cellular extracts from GFP-expressing parasites. GFP: wild-type promastigotes. SKO-GFP-2.1 and SKO-GFP-6.1: Two independent GFP-expressing *LiEndoG^−/+^* promastigote clones. A Polyclonal α-Hsp70 (1∶5000) antibody was used as a loading control. **B**) and **C**). Intracellular infections. PMA-differentiated THP-1 cells were infected with promastigotes expressing the green-fluorescent protein (eGFP). **B**). Percentages of infection were evaluated by counting the number of green-fluorescent PMA-differentiated THP-1 cells by flow cytometry. **C**) Parasite load was determined based on the mean fluorescence intensity (arbitrary units; AU) of the infected THP-1 cells. **D**). Analysis of mitochondrial membrane potential based on the relative fluorescence due to Mitotracker Red CMXRos. Error bars symbolize standard deviations; results are representative of three independent experiments. The mean value of the clones differ from that of WT with p values <10^−3^(*), <10^−4^(**), <10^−5^(***).

LiEndoG-deficient parasites were also tested for their ability to grow inside infected macrophages. Clones 2, 6 and wild-type parasites were transfected with a plasmid containing the sequence coding for eGFP. After incubation with nourseothricin and selection of two random clones (SKO-GFP-2.1 and SKO-GFP-6.1), the fluorescent parasites were incubated with differentiated THP-1 cells and both the infection rates and the mean fluorescent values of the infected cells were evaluated by flow cytometry. The number of infected macrophages is significantly reduced when incubating with SKO-GFP-2.1 and SKO-GFP-6.1 clones (Figure 4B). Moreover, the parasite load inside the infected THP-1 cells is also strongly decreased as indicated by the much lower mean fluorescence intensities of the infected cells (Figure 4C). Accordingly, LiEndoG-deficient parasites also have growth defects during macrophage infection, confirming the pro-survival function of this protein in amastigotes.

Because of the mitochondrial location of this protein, we also evaluated any possible disturbances in the morphology of this organelle in the *LiEndoG* ^−/+^ clones but no significant differences could be observed. However, the LiEndoG-deficient parasites show increased Mitotracker-derived fluorescence intensities when compared with WT ones (Figure 4D), which demonstrates an increased mitochondrial transmembrane potential (Δ*ψ*m) in the *LiEndoG* ^−/+^ clones. This constitutes an unexpected result as LiEndoG deficiency is expected to impair the mitochondrial function. However, it has already been demonstrated that DNA damages in the mitochondrial DNA of *Leishmania* parasites cause an increase in their Δ*ψ*m [19].

**Figure 5:**
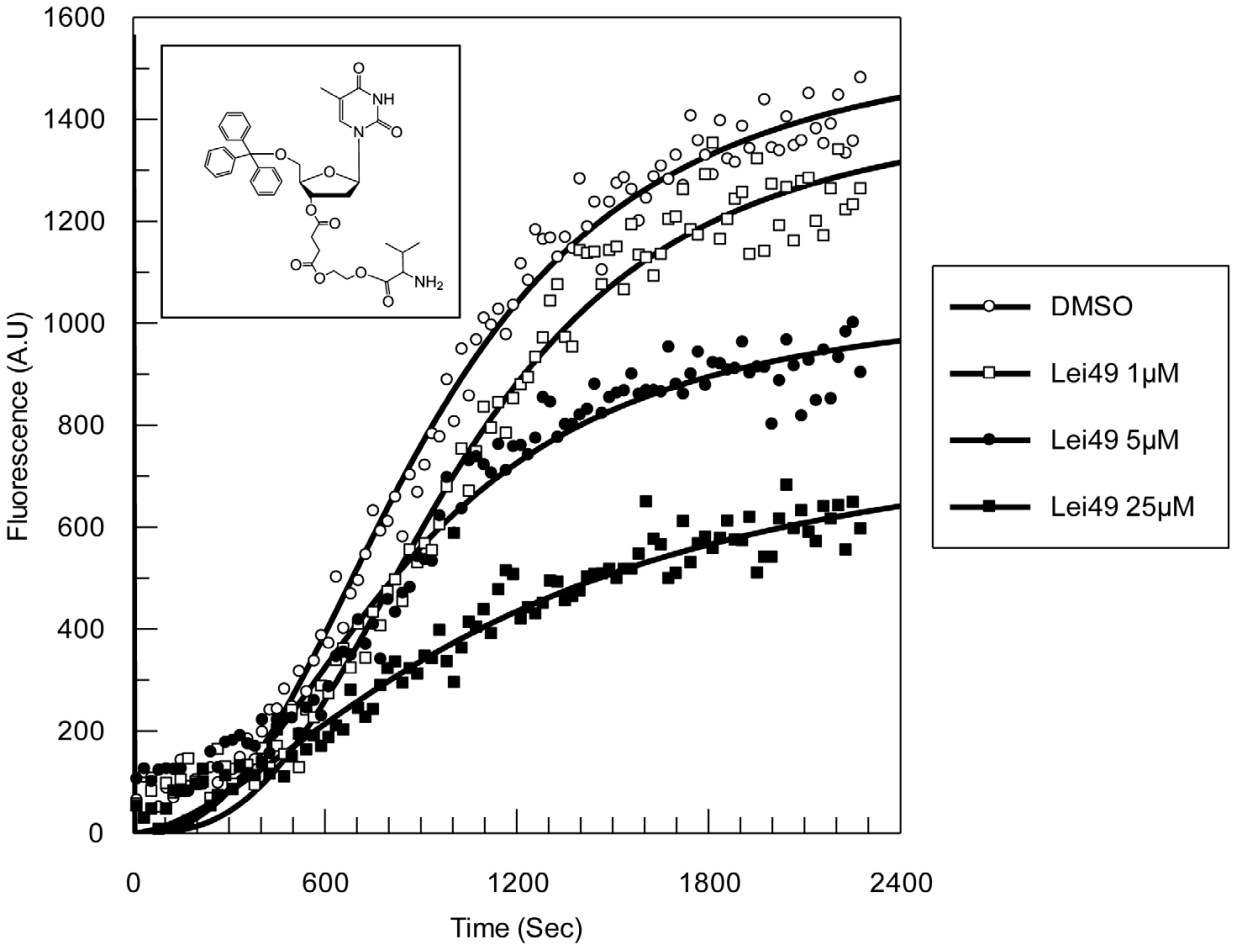
Inhibition of the nuclease activity of LiEndoG by Lei49. LiEndoG activity was assayed by the cleavage of a dually labeled double-stranded probe. DNA degradation is followed by the increase in fluorescence observed over 40 min of probe digestion in the presence of increasing amounts of **Lei49**.

**Figure 6:**
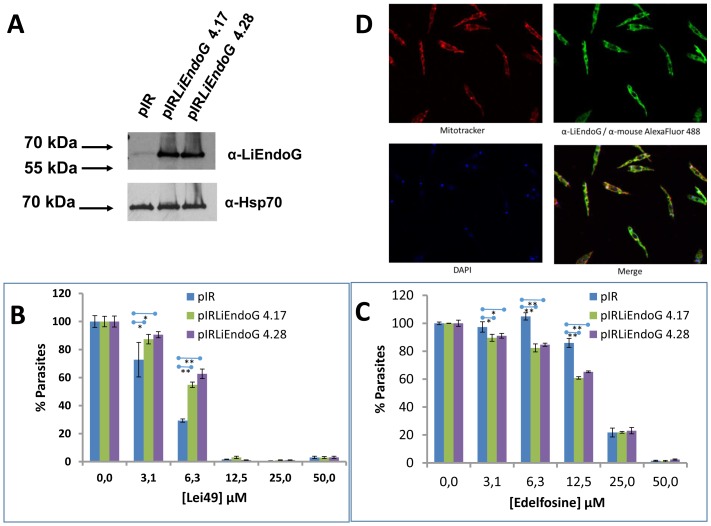
Drug-induced cell death in parasites overexpressing LiEndoG. **A**) Immuno-blot using the monoclonal antibody α-rLiEndoG against cellular extracts from parasites transfected with an empty vector (pIR) and from two independent clones overexpressing LiEndoG (pIR*LiendoG* 4.17; pIR*LiendoG* 4.28). A polyclonal α-Hsp70 (1∶5000) antibody was used as a loading control. **B**) Percentages of PI negative (alive) cells in the three cell lines treated with increasing concentrations of **Lei49**. **C**) Percentages of PI negative (alive) cells in the three cell lines treated with increasing concentrations of Edelfosine. Error bars symbolize standard deviations; results are representative of three independent experiments. **D**) Mitochondrial localization of LiEndoG in the overexpressing clone pIR*LiendoG* 4.28. Similar results (not shown) were obtained for clone pIR*LiendoG* 4.17. The mean values of the clones differ from that transfected with an empty vector with p values <10^−1^ (*) or <10^−2^ (**).

We have recently identified several molecules able to reduce the activity of LiEndoG *in vitro* [10]. Among them, **Lei49** behaves as one of the best inhibitors (Figure 5) and also as one of the compounds with better cytotoxic activity against *L. infantum* promastigotes (IC_50_ = 4.33±0.05 µM) and amastigotes (IC_50_ = 6.44±0.01 µM). To demonstrate that LiEndoG activity is essential for parasite survival, **Lei49**-induced death rates were analyzed in control parasites transfected with an empty vector and in parasites overexpressing LiEndoG (Figure 6A). Parasite viability upon treatment with increasing concentrations of **Lei49** or edelfosine was measured based on the percentage of propidium iodide (PI) negative cells. The results presented in Figure 6B demonstrate that parasites overexpressing this enzyme are significantly protected against cell death caused by moderate concentrations of **Lei49** but, as expected, increasing concentrations of the compound abolish the protective effect of overexpression. By contrast, as previously described [3], the same parasites are more susceptible to edelfosine-induced cell death (Figure 6C), probably as a consequence of increased activity of the pro-apoptotic molecular machinery. As shown in Figure 6D the overexpressed protein, similarly to the endogenous one, is correctly targeted to the mitochondrion. These results support the relevance of LiEndoG for parasite survival in addition to its better known pro-death role.

## Discussion

We show that LiEndoG is an endo-exonuclease that is able to generate single-strand breaks in circular supercoiled DNA whereas it has a preferential 5′ exonuclease activity over linear DNA. Previous findings from us and from other groups have already demonstrated that this nuclease activity is relevant during the apoptotic cell death process of the parasites [3], [20], [21]. The results presented herein demonstrate that this enzyme is also essential for survival. Firstly, we show that parasites which, as a consequence of single allele replacement, contain reduced levels of LiEndoG protein grow at much lower rates than do their wild-type counterparts. Secondly, we demonstrate that these LiEndoG-deficient parasites are much less infectious for macrophages. Thirdly, we prove that the cytotoxic activity of the LiEndoG inhibitor **Lei49** is significantly reduced when the protein is overexpressed in the parasites. Our results agree with those reported by Buttner et al. who showed that deletion of Nuc1p reduces yeast growth rates, an effect attributed to its possible role in mtDNA recombination [9]. Indeed, orthologs of EndoG in *S. cerevisiae* and in *Neurospora* initiate homologous recombination by generating single-stranded regions in the chromosomes by means of their 5′ exonuclease activity [22], [23]. The 5′ exonuclease activity described for LiEndoG in this article could be considered as an indication of its possible involvement in recombination and repair of mitochondrial DNA, even though more work will be necessary to prove this point. Taken together, our results strongly indicate that LiEndoG does indeed play a dual pro-life and pro-death role in *L. infantum* similar to that described for the EndoG orthologs in yeast and *C. elegans*. Moreover, the involvement of LiEndoG in the survival of these parasites makes this protein a potential target against which new leishmanicidal drugs could be developed.
